# Associations of Physical Activity, Muscle Mass and Protein-Rich Food Consumption with Functional Fitness in Individuals with Multiple Sclerosis

**DOI:** 10.3390/nu18101548

**Published:** 2026-05-13

**Authors:** Elżbieta Cieśla, Elżbieta Jasińska, Edyta Suliga

**Affiliations:** Department of Health Sciences, Jan Kochanowski University, 25-369 Kielce, Poland; elzbieta.jasinska@ujk.edu.pl (E.J.); edyta.suliga@ujk.edu.pl (E.S.)

**Keywords:** multiple sclerosis, sarcopenia, physical activity, protein-rich foods, handgrip strength, manual dexterity

## Abstract

Background: Multiple sclerosis (MS) is a chronic disease of the central nervous system, characterised by high variability in both its progression and symptoms. The disease leads to progressive disability, which manifests itself as slow walking, low muscle mass and impaired manual dexterity, causing difficulties in performing everyday activities and reducing a patient’s social activity and quality of life. The aim of this study is to assess the relationships between muscle mass, physical activity and the food frequency of protein-rich products and the functional fitness of patients with MS. Methods: The study comprised 106 patients with MS (83 women and 23 men) aged 18–65 years. Measurements of their weight and body composition, motor function of the lower limbs using the Timed 25-Foot Walking Test (T25FW), and motor function of the upper limbs using the 9-Hole Peg Test (9-HPT) and the Handgrip Strength (HGS) test were performed. Daily moderate-to-vigorous physical activity (MVPA) and the consumption frequency of protein-rich products were also assessed. Results: Low muscle mass was associated with worse performance in the HGS test (non-dominant hand *p* = 0.001, dominant hand *p* = 0.001), while no significant associations were observed for manual dexterity or T25FW performance.. The second tercile of MVPA was significantly associated with reduced HGS in the dominant (*p* = 0.037) and non-dominant hands (*p* = 0.015). Conversely, the third tercile of the MVPA compared to the lower tertile was associated with better HGS of the non-dominant hand (*p* = 0.022) and faster completion of the 9-HPT with the non-dominant (*p* = 0.010) and dominant hands (*p* = 0.029). Furthermore, frequent consumption of protein-rich products was correlated with faster completion of the T25FW test (*p* = 0.033). Conclusions: Regular physical activity is associated with better functional fitness, while more frequent consumption of protein-rich foods may be associated with higher muscle mass of major muscle groups, which is important for effective locomotion. This study has a cross-sectional and exploratory design; therefore, the findings reflect associations only and do not allow casual inferences.

## 1. Introduction

Multiple sclerosis (MS, from the Latin *sclerosis multiplex*) is a chronic, autoimmune degenerative disease. Its varied clinical manifestation is related to the number, volume and location of the demyelinating changes occurring within the central nervous system. Growing damage to the brain and/or brain stem leads to a progressive disability involving, among other effects, reduced muscle mass, increased muscle tension and coordination disorders [1]. The resulting impaired functioning makes it difficult for patients to perform everyday activities and limits their participation in social life, thus reducing their quality of life [2]. Research indicates that in the first decade of MS, as many as 90% of patients experience fatigue and sensory disorders. Likewise, 78% report difficulties with their mobility, cognitive functions and manual dexterity, while 71% report shaking and impaired coordination [3]. 

The motor dysfunctions observed in patients with MS result from damage to the central nervous system and are exacerbated by insufficient physical activity and a progressive loss of muscle strength [4]. Regular physical activity is a key element in maintaining one’s overall health, including in patients with MS, and structured exercise regimes in persons with mild or moderate disability may considerably improve their aerobic capacity, muscle strength and gait parameters [5]. However, despite the documented benefits, an unwillingness to exercise is common among patients with MS and intensifies along with the disease [6,7,8]. 

Low physical activity is a frequent consequence of the symptoms typical for MS, such as progressive disability and chronic fatigue, which promote an increased share of sedentary behaviour [9].

An earlier study showed that structured exercise regimes lasting 8–24 weeks carried out at least 2–3 times a week that include aerobic training, resistance training and balance exercises lead to an improvement in aerobic capacity, muscle strength, functional fitness and fatigue resistance in individuals with mild to moderate disability [5].

Furthermore, a later study demonstrated that different types of training provide different benefits. Aerobic and interval training improves walking efficiency and locomotor control, whereas resistance training contributes to an increase in muscle strength [10]. In addition, at-home exercise regimes involving task-based and proprioceptive training are associated with improved upper limb function, including HGS [11].

Despite the well-documented benefits provided by structured exercise regimes, only a relatively small percentage of individuals with MS meet the recommended volume of physical activity (≥150 min per week), which includes both supervised exercise and lifestyle-related activity [6]. Moreover, the latest evidence suggests that the effects of training interventions do not always translate into an increase in habitual physical activity in everyday life [12].

According to the European Working Group on Sarcopenia in Older People (EWGSOP), the basic diagnostic criteria for sarcopenia are age-related or chronic disease-related losses of lean body mass combined with low muscle strength [13]. The available data indicates a varied share of MS patients who meet the criterion for sarcopenia, as determined by age and the degree of disability assessed using clinical scales [14,15,16]. The relationships between reduced muscle mass, which is a fundamental diagnostic criterion for sarcopenia, and functional fitness in patients with MS have not been sufficiently investigated, and the available data is contradictory. Stagsted et al. observed an indirect role of muscle mass in patients’ motor functions, because lower limb strength, which depends on muscle mass, correlated significantly with the results of the T-25FW test [17]. Conversely, Jeng et al. did not find statistically significant relationships between body composition and motor functions, even after taking the degree of disability into account. This suggests that mass and fat parameters alone may be insufficient predictors of a person’s walking functions [18]. However, studies on sarcopenia in patients with MS indicate that extremely low muscle mass, falling below established diagnostic criteria, is associated with a marked decrease in a patient’s mobility parameters and reduced muscle strength [14,16]. The literature underlines a need for further research on this topic [14]. In addition, no clear relationships between manual dexterity and reduced muscle mass in patients with MS have been identified to date [14,19].

The consumption of complete protein is a key factor in preventing loss of muscle mass and the development of sarcopenia [20]. However, the optimal amounts and types of protein have yet to be determined, especially with respect to neurological diseases, such as MS. 

The literature lacks studies involving a comprehensive analysis of the relationships between sarcopenia, consumption frequency of protein-rich products and the level of physical activity, and their combined effect on functional fitness, as assessed based on HGS, manual dexterity and gait parameters. The research conducted so far has focused exclusively on selected associations, such as the relationships between physical activity and functional fitness, between sarcopenia and functioning, or between diet and body composition, while ignoring the potential synergistic actions of these factors [4,5,6]. The inclusion of their combined effect on the population of patients with MS is particularly important due to the complexity of MS symptoms. 

The aim of this study is to assess the relationships between the level of physical activity, muscle mass and consumption frequency of protein-rich products and the HGS, manual dexterity and walking function in persons with MS. 

## 2. Materials and Methods

### 2.1. Population

The study was conducted between October 2021 and January 2022. A total of 130 patients with MS were assessed for eligibility, of which 106 participants who met the inclusion criteria were selected (83 women, 78.30%; 23 men, 21.70%). The mean age of the participants was 42.36 ± 11.54 years, and the mean duration of diagnosed MS was 10.27 ± 8.97 years. 

The diagnosis of MS in the participants was confirmed according to the McDonald criteria [21]. A vast majority of the participants showed relapsing–remitting multiple sclerosis (RRMS; *N* = 89, 89.12%). Only 7.92% were diagnosed with secondary progressive MS, with the remaining 3.96% diagnosed with primary progressive MS. Patients aged 18–65 years with an Expanded Disability Status Scale (EDSS) score of < 7 points qualified to participate in the study [22]. Persons with an EDSS score ≥7 points (i.e., requiring mobility aids such as wheelchairs), as well as those with additional serious central nervous system disorders (e.g., stroke, intracranial haemorrhage, tumours or myelitis), were excluded. None of the participants had used glucocorticosteroids in the six months preceding the study. 

### 2.2. Anthropometric Measurements and Body Composition Assessment

Height was measured using an SECA stadiometer with an accuracy of 0.1 cm. Weight and body composition were measured using a Tanita MC-780MA-N analyser. The analysis section presents the parameters related to fat-free mass and muscle mass for the whole body and its individual segments. In addition, the BMI was calculated using the following formula: BMI = weight [kg])/height^2^ [m^2^]. The obtained values were categorised as follows: normal BMI (<25.0 kg/m^2^) and overweight and obesity (≥25.0 kg/m^2^). 

### 2.3. Sarcopenia Assessment

Sarcopenia was assessed based on the ratio of the Appendicular Skeletal Muscle Index (ASMI) calculated as appendicular muscle mass divided by height squared (kg/m^2^). Appendicular skeletal muscle mass, used to calculate ASMI, was estimated using bioelectrical impedance analysis (BIA) with a Tanita MC-780MA-N analyser. Separate cut-off points were used for men (<7 kg/m^2^) and women (<5.5 kg/m^2^) [13]. Based on ASMI values, participants were classified into two groups: those with sarcopenia (*n* = 25) and those with normal muscle mass (*n* = 81). 

### 2.4. HGS Measurement

Dominant and non-dominant handgrip strength (HGS) was measured in a seated position, with the participant’s elbow flexed at 90°, using a Kyto Hand Dynamometer (maximum force 290 lbs/90 kg). Each participant performed two trials with each hand, and the results were averaged separately for the dominant and non-dominant hand. The averaged values and the sum of HGS measurements for both hands were used in the analyses. In our research, the intraclass correlation coefficients (ICC) demonstrated excellent reliability of repeated handgrip strength measurements (right hand: ICC = 0.95; left hand: ICC = 0.91). Persons with multiple sclerosis tend to experience increased muscle fatigue, which may affect the results of repeated measurements [17]. For this reason, the number of trials was reduced to two. An average value was applied, rather than the best result, in order to obtain a more stable and representative measure of muscle strength [23].

### 2.5. Manual Dexterity Assessment

Manual dexterity was assessed using the 9-HPT, which involves inserting nine pegs into nine holes, one by one, and then taking them back out of the holes and putting them in a bowl. The measured parameter was the time of completion. The test was performed twice for each hand. The averaged completion time, calculated separately for each hand, was used in the subsequent analyses [2].

### 2.6. Assessment of Motor Functions of the Lower Limbs

The motor function of the lower limbs was assessed using the Timed 25-Foot Walk test (T25FW), which involves walking a distance of 25 feet (7.6 m) as fast as possible while maintaining safety. Each participant performed the test twice, and the results were averaged [24].

### 2.7. Physical Activity Measurement

Daily physical activity was measured using an ActiGraph GT3X-BT accelerometer, which participants were instructed to wear on the non-dominant wrist for up to eight consecutive days. Participants recorded the times when the accelerometer was removed and reattached each day. Data analysis included only days with at least 10 h of valid wear time. Wearing an ActiGraph on the wrist is well tolerated by persons with MS and yields activity patterns comparable to those obtained from ankle measurements while also recording upper limb movements [25].

Accelerometer data were recorded in 10-second epochs at a sampling frequency of 30 Hz. Periods of ≥60 consecutive minutes with no recorded movement were classified as non-wear time. The following parameters were analysed: percentage of time spent in light physical activity (LPA), moderate-to-vigorous physical activity (MVPA) and sedentary behaviour (SB). Based on MVPA percentage, participants were categorised into three tercile-based groups: low (T1), medium (T2), and high (T3). The cut-off values for MVPA were 12.8% for T2 and 17.08% for T3.

### 2.8. Assessment of the Consumption Frequency of Protein-Rich Products

Consumption frequency was determined using the KomPAN questionnaire [26]. The questionnaire included questions about the consumption of dairy products (kephir, natural yoghurt, cottage cheese and hard cheese), cold cuts, sausages, red and white meat, fish and legumes. Consumption frequency was divided into the following categories: never, 1–3 times per month, once per week, several times per week, once per day and several times per day. The categories were transformed into units/day as suggested in the questionnaire: never = 0; 1–3 times per month = 0.06; once per week = 0.14; several times per week = 0.5; once per day = 1; several times per day = 2, and total.

### 2.9. Statistical Analysis

The sample size was determined using a power analysis. Assuming a moderate effect size (*r* = 0.35), significance level (*α* = 0.05) and power level (0.80), the minimal sample size required to detect an effect was 62 persons. 

Qualitative variables were described as numbers (*N*) and percentages in the groups of participants with reduced and normal muscle mass. Between-group relationships were investigated using a non-parametric chi-squared test. Quantitative variables were described as the means ± standard deviation (*X* ± *SD*) or as the medians and interquartile ranges (*Me*, IQR). Student’s *t*-test or the Mann–Whitney *U* test was used depending on the distribution of characteristics within the groups.

Generalised linear models with an identity link function analysis were used to assess the effects of MVPA, ASMI and consumption frequency of protein-rich products on the aspects of functional fitness, with T25FW, muscle strength and 9-HPT (the right and the left hands were analysed separately) as the dependent variables. Each model also included sex and EDSS as covariates. Additionally, multiple statistical models were tested without formal correction for multiple comparisons, which may increase the risk of type I error. Therefore, the findings should be interpreted as exploratory.

Model fit measures and tests verifying regression assumptions (normality of residual distribution, heteroscedasticity and collinearity) were calculated for each model. The results were considered statistically significant at *p* < 0.05. The analyses were performed in the STATISTICA 13.3 software package.

### 2.10. Ethical Aspects

The study was approved by the Scientific Research Ethics Committee of the Medical College at the Jan Kochanowski University in Kielce, Poland (Approval No. 24/2020 from 25 April 2020). The study involved collecting data on the participants’ socioeconomic conditions, sociodemographic information, age of MS diagnosis, medicines used and comorbidities. All the measurements were conducted by qualified medical personnel (nurses), in accordance with the established protocol and standard measurement techniques.

## 3. Results

The group with a low ASMI showed no significant relationships between the type of MS, age, length of MS, degree of disability or socioeconomic status, and the ASMI (Table 1). Physical activity and sitting behaviour were also similar in both groups. The HGS in both hands and the total HGS were significantly lower in the group with a low ASMI than in the group with a normal ASMI. Conversely, the results of the T25FW test and 9-HPT for both hands and body fat percentage (FatP) were identical between the two groups. Fat mass in kg (FatM), predicted muscle mass (PMM) and fat-free mass (FFM) assessed for the whole body and its individual segments were significantly lower in the group with a low ASMI. The consumption frequency of protein-rich products was similar in both groups (Table 1). 

**Figure 1:**
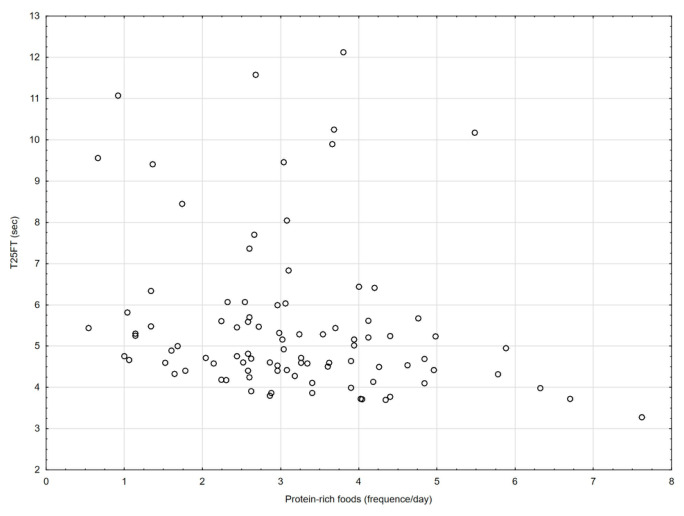
Association between consumption of protein-rich foods and T25FW.

**Figure 2:**
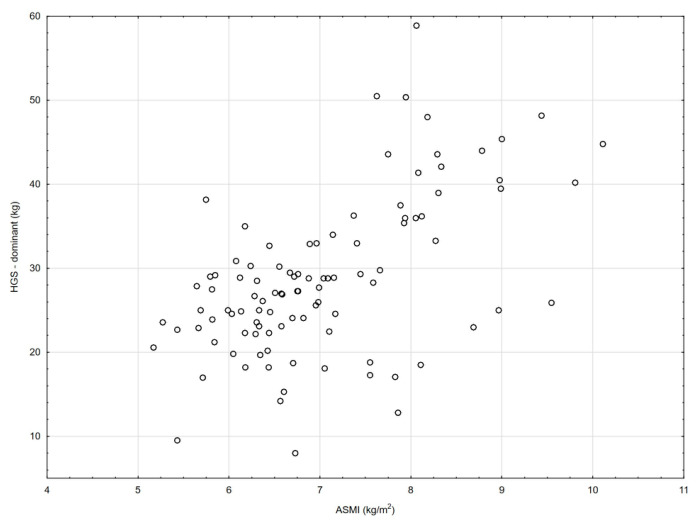
Association between ASMI and dominant handgrip strength (HGS).

**Figure 3:**
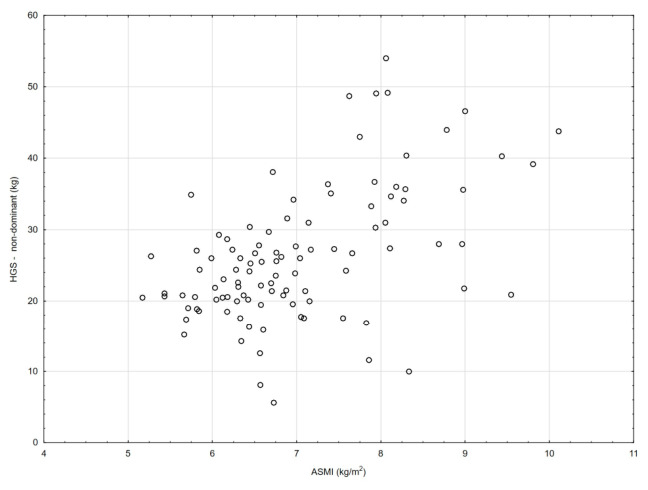
Association between ASMI and non-dominant handgrip strength (HGS).

**Figure 4:**
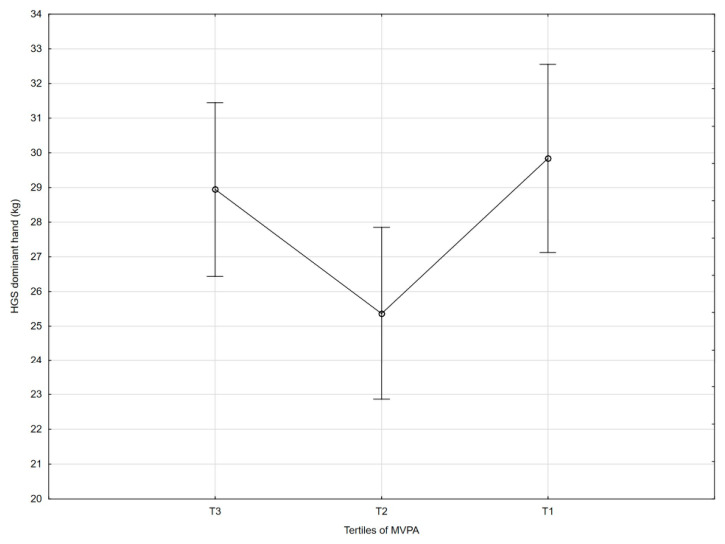
Association between MVPA and dominant handgrip strength (HGS).

**Figure 5:**
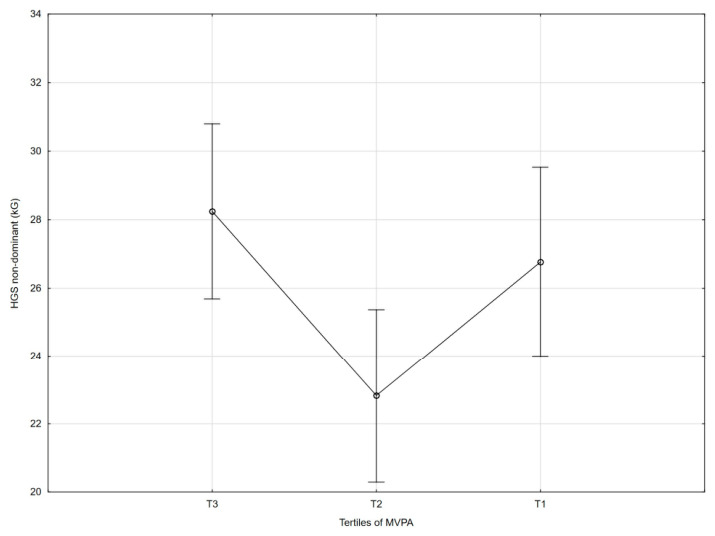
Association between MVPA and non-dominant handgrip strength (HGS).

**Figure 6:**
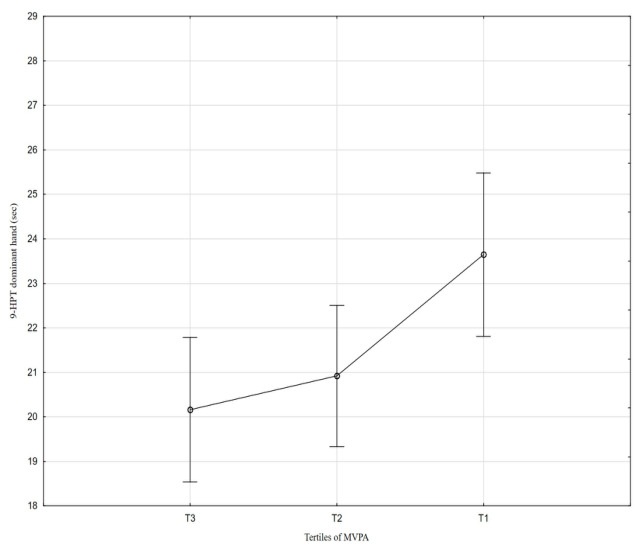
Association between MVPA and 9-HPT performance in the dominant hand.

**Figure 7:**
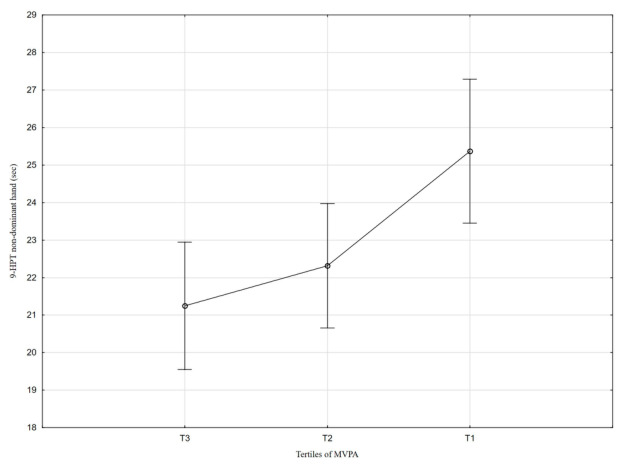
Association between MVPA and 9-HPT performance in the non-dominant hand.

Table 2 and Figure 1, Figure 2, Figure 3, Figure 4, Figure 5, Figure 6 and Figure 7 show the relationships between the ASMI, MVPA and consumption frequency of protein-rich products and the characteristics of functional fitness in persons with MS. The ASMI was positively associated with muscle strength parameters, indicating that lower ASMI values corresponded to lower levels of selected motor function measures (dominant hand: *β* = 4.57; *p* = 0.001; non-dominant-hand: *β* = 3.88; *p* = 0.001). Patients with medium MVPA (second tercile) compared to patients with MS with low MVPA (first tercile) showed significantly lower HGS (dominant hand: *β* = −2.13, *p* = 0.037; non-dominant hand: *β* = −3.11, *p* = 0.015). Physical activity in the third tercile significantly improved parameters of HGS and manual dexterity in the non-dominant hand (HGS: *β* = 2.30, *p* = 0.022; 9-HPT: *β* = −1.73, *p* = 0.010) and manual dexterity in the dominant hand (*β* = −1.41; *p* = 0.029). The frequency of consumption of protein-rich products was weakly associated with T25FW (*β* = −0.22, *p* = 0.033), indicating shorter completion time and thus better walking performance; however, this finding should be interpreted with caution.

## 4. Discussion

### 4.1. Low Muscle Mass and MS

The results show an association between low muscle mass and lower HGS, but no significant association was found between low muscle mass and T25FW and manual dexterity assessed using the 9-HPT. To date, few studies have analysed the relationships between reduced muscle mass and functional fitness in patients with MS [27]. Even so, the available reports suggest that the individual indicators of muscle mass, as well as a full diagnosis of sarcopenia, are related to a low level of functional fitness [14,16]. However, there are some inconsistencies. Pilutti and Motl observed that a positive relationship between FFM and fitness only concerned HGS, whereas the T25FW test did not correlate with FFM in persons with moderate disability [28]. According to some papers, the primary factor is muscle mass in the lower limbs rather than the total muscle mass [18,29]. FFM is the leading factor determining muscle mass, and a lack of mutual relationships has also been confirmed in other research projects [30].

Our results support the lack of a clear relationship between reduced muscle mass and 9-HPT performance and T25FW. Because the 9-HPT reflects a person’s dexterity and precision, it may be insensitive to reduced muscle mass at an early stage of sarcopenia [31]. Similarly, T25FW test performance may not be directly related to overall muscle mass, as walking ability in persons with MS depends on multiple factors, including neuromuscular function, balance, fatigue, and disease severity. Therefore, total muscle mass alone may not be a sufficient indicator of walking performance [23].

A study by Yuksel et al. demonstrated that persons with MS and sarcopenia displayed reduced HGS and walking parameters compared to both healthy persons and persons with MS but without sarcopenia [14]. Similar relationships were found in older patients with MS, in whom a reduced muscle mass was associated with reduced muscle strength [16]. These results suggest that lower muscle mass is a significant problem in both the adult and elderly populations of persons with MS that may be associated with poorer functional fitness. A populational analysis conducted by the UK Biobank showed a more rapid drop in muscle strength than muscle mass in persons with MS, which suggests a potential contribution of demyelination, muscle activation disorders and a limited recruitment of motor units in the development of sarcopenia in this population [15]. Earlier studies also demonstrated that sarcopenia may be more prevalent in persons with MS [14,16]. Structural changes within the nervous system, neuromuscular conductivity disorders and the other symptoms observed frequently in persons with MS, including intensified physical fatigue, may be associated with walking deficits and reduced limb strength, even though they usually do not yet affect precise hand movements at an early stage.

Reduced muscle mass in persons with sarcopenia but without MS occurs first in the large muscle groups of the lower limbs. Consequently, patients with MS may be particularly at risk of a loss of muscle mass and functional fitness. Further decreases in muscle strength and walking functionality may be related to the changes in the structure of the nervous system, neuromuscular conductivity disorders, increasingly frequent physical fatigue, low physical activity and the sedentary lifestyle that accompany MS [32]. Our findings support this potential sequence of function loss: large muscle groups weaken first, affecting walking and HGS, while manual dexterity remains relatively intact. Although this study did not directly evaluate lower limb strength, earlier works suggested that HGS is a reliable marker of overall muscle strength [33]. The loss of HGS may also suggest processes that affect other muscle groups, specifically, decreased muscle cross-sections, loss of motor units and conductivity disorders. A study by Seferoğlu et al. showed that reduced HGS in persons with MS was related to impaired balance, weakened lower limb functions and a decreased quality of life [34].

### 4.2. Physical Activity and MS

Moderate-to-vigorous physical activity was not significantly associated with the T25FW score, whereas the relationship between MVPA and other functional parameters appeared non-linear, which may be explained by variability between participants and other factors not included in the analysis. In particular, the level of activity in the second tercile was insufficient to improve HGS, whereas the highest level of activity (third tercile) was associated with both higher strength and better 9-HPT performance. The tercile analysis demonstrated that the third tercile accounted for ≥17.08% of the daily MVPA. With an average period of 10 h of wearing the accelerometer each day, this translates into about 1.1 h of moderate daily activity. 

The observed non-linear pattern of this relationship may be explained by variability between participants and other factors not included in the analysis, as well as, to some extent, by the method used to assess physical activity. In this study, physical activity was measured using wrist-worn accelerometers, which may reflect upper limb activity to a greater extent than overall locomotor activity. Therefore, this result should be interpreted with caution.

The obtained results partially overlap with earlier cross-sectional and intervention studies on the relationship between physical activity and fitness in persons with MS. Baird et al. observed that the relationship between physical activity and T25FW test performance only appears when the confounding variables are controlled, including the age, length of MS, race and ambulatory disability [35], which suggests that physical activity may support the maintenance of mobility, especially in older persons. Walking, which is normally classified as light physical activity, likely becomes moderate among this group, which would explain the stronger relationships observed in seniors. The study also found a strong negative correlation between MVPA and completion time of the T25FW test. However, this association was only present in an older group of adults (60–79 years), which suggests that physical activity may be associated with better maintenance of walking functionality, especially at an older age. Motl et al. also observed a clear relationship between total physical activity and T25FW test performance [36]. However, it is worth underlining that in this case, the authors measured not only the MVPA, but also overall daily physical activity, which implies that total activity has a significant relationship with the T25FW test. Some studies assessing gait speed using the T25FW test and other tests only reported moderate associations with daily physical activity [37]. A lower correlation may result from the clinical characteristics of persons with MS: spasticity, balance and sensory disorders, fatigue and fear of falling, and additional environmental conditions. Although a person with MS may walk faster during a short test, they may not be able to maintain that speed in real, everyday conditions. Our results also partially correspond to those obtained by Feasel et al., who observed the lack of a relationship between aerobic reserve capacity and short-distance gait speed [38]. Furthermore, a three-year observation of patients with MS and mild disability did not reveal any changes in the T25FW test performance or the level of daily physical activity, suggesting a lack of co-variation between these two parameters [39]. Even though the T25FW is considered a sensitive and reliable test of lower limb strength, it measures the short-term gait speed rather than overall physical activity. The parameter is sensitive to factors such as EDSS, age or the MS phenotype. Consequently, the T25FW may not always reflect the overall level of physical activity [24].

A meta-analysis conducted by McManaman et al. showed that a structured strength training regime lasting from between 6 and 24 weeks, regardless of the exercise volume, significantly improved the gait speed in persons with MS compared to controls [40]. This implies that regular resistance activity may affect motor functions through neuromuscular mechanisms, rather than solely by increasing the muscle mass. With respect to the upper limb functions, there is evidence that physical activity measured with an accelerometer is moderately and negatively correlated with the results, which is consistent with the finding that a better 9-HPT outcome is related to a shorter completion time [41]. In a study conducted by Lamers et al., the upper limb activity registered with a wrist accelerometer correlated negatively with the 9-HPT time for the non-dominant hand, which suggests that frequent use of the upper limbs in daily activities indicates a high level of manual dexterity [42].

The number of studies assessing the relationship between physical activity measured with an accelerometer and the 9-HPT performance remains low, primarily due to methodological differences in the location of the sensors. Hip-worn or belt-worn accelerometers primarily register locomotor activity (T25FW test and 6-Minute Walk Test), which explains the lack of significant associations with manual dexterity tests [43,44,45]. However, studies that assessed the upper limb functions (including HGS and 9-HPT), in which the sensors were mounted on the wrists, were able to record significant relationships. Persons who were more active in their daily life achieved shorter 9-HPT completion times and higher HGS [41,46]. Thus, a relationship between physical activity and the 9-HPT is plausible. Even though the test primarily assesses distal movements, performing it also requires proximal stabilisation and activation of the arm muscles. However, it is worth noting that some studies did not confirm a relationship between MVPA and the number of steps taken, which suggests that the level of physical activity does not always directly translate into mobility [12].

Regular physical activity may support the improvement in neuromuscular mechanisms by increasing neuroplasticity in the central nervous system and the recruitment of motor units, which is supported by neuroimaging tests that indicated the activation of grey matter and a reorganisation of the motor cortex after motor training [47,48,49]. Higher physical activity correlates with the volume of grey matter and deep brain structures, while increased aerobic capacity is associated with a higher volume of limbic and subcortical structures [50,51]. Furthermore, there is evidence that regular physical activity and exercise may have a neuroprotective effect and may be associated with neural plasticity in patients with MS [52]. EMG assessments suggested that long-term resistance induced neuromuscular adaptations that persisted even over a 24-week period of activity. According to a meta-analysis conducted by Cruickshank et al., strength training in patients with MS is related to a significant alleviation of chronic fatigue syndrome, along with improved functional fitness, quality of life and electromyographic power and activity [53]. These results further corroborate the thesis that physical activity is associated with muscle functions and may be associated with improved performance in manual tests, including the 9-HPT. Furthermore, reduced oxidative stress and C-reactive protein (CRP) levels are observed following both individual activities and long-term interventions [54]. 

### 4.3. Intake of Dietary Protein-Rich Foods and MS

Dairy products provide a range of nutrients and bioactive compounds that benefit the regeneration and health of muscles [55]. Dairy proteins were found to strongly stimulate post-exercise muscle anabolism, promoting lean body mass gain in populations of healthy middle-aged and elderly persons [56]. In other clinical populations (such as elderly persons with neurodegenerative disorders), the regular consumption of dairy products was also associated with cognitive benefits [57]. Similarly, red meat contains a number of micro- and macronutrients important for the functioning of the nervous and muscle systems, and a deficiency in these nutrients is related to a higher risk of MS and its progression [58,59]. Particularly important are heme iron, long-chain omega-3 fatty acids, B vitamins, and vitamin D, which are linked to muscle regeneration and the functioning of large muscle groups, and may be associated with walking performance [60,61,62,63]. 

A borderline association was observed between the frequency of protein-rich food consumption and T25FW performance; however, this finding should be interpreted with caution and requires confirmation in larger samples. Conversely, we did not observe an effect of diet on performance in other functional fitness tests involving precise hand movements. These results may suggest a potential association between dietary factors and muscle function in individuals with MS. The lack of a relationship between the consumption of the aforementioned products and the results of other fitness tests may be related to the fact that such results pertain mostly to precise hand motions, and consequently, to the involvement of small muscle groups. 

In our review of the literature, we did not find direct evidence of a relationship between the consumption frequency of protein-rich products and the performance in physical fitness tests (T25FW, 9-HPT and HGS) in persons with MS. Nonetheless, observations of other populations suggest a potential association between protein intake and muscle mass and strength [64,65]. A supplementation of dairy protein in middle-aged and elderly persons with sarcopenia was associated with improvements in muscle mass of the limbs, while the consumption of milk and yoghurt was correlated with HGS [66]. A study conducted by Mazza et al. demonstrated that adherence to a Mediterranean diet, rich in legumes, grains, fruits and vegetables, with limited amounts of meat, fish and eggs, correlated positively with HGS and skeletal muscle mass and was linked to the lowest risk of low muscle strength and sarcopenia [67]. However, the participants of Mazza et al.’s study did not include persons with MS. We were unable to find studies that unambiguously indicated a positive relationship between the frequent consumption of meat and dairy products with muscle mass in persons with MS. 

Immunological mechanisms related to some milk proteins may be associated with an auto-inflammatory response against myelin and a higher risk of MS or differences in the course of the disease [68]. In a study of persons with MS, excessive consumption of meat and dairy products was related to a higher degree of disability or a higher risk of MS progression [69]. However, after factors such as age and sex were included, such relationships were often found to be statistically insignificant [64]. An important finding was that the frequent consumption of meat and dairy products did not necessarily mean a higher intake of protein, because FFQs do not evaluate the real quantitative consumption. On the other hand, consumption frequency may reflect a more regular replenishment of the amino acids and micronutrients that are key to muscle regeneration. 

### 4.4. Limitations and Strengths

Our study had certain limitations. Firstly, most of the patients included in the study represented RRMS, which made it difficult to generalise the results over the other MS phenotypes and prevented the determination of causal relationships. Additionally, multiple statistical models were tested without formal correction for multiple comparisons, which may increase the risk of type I error. Therefore, the findings should be interpreted as exploratory.

The study also did not take into account the level of fatigue, which may be linked to physical activity and the parameters of functional fitness. This, in turn, limited us in obtaining a full understanding of the relationships between the participants’ consumption of protein-rich products, physical activity, ASMI and motor functions. Furthermore, dietary intake was assessed using a food frequency questionnaire (FFQ) focused on protein-rich products, which reflects the frequency of consumption rather than precise quantitative intake and therefore limits the strength of conclusions regarding the relationship between diet and functional outcomes. Another limitation of this study is that the observed associations between the frequency of consumption of protein-rich foods and functional outcomes were weak and of borderline significance. Therefore, these findings should be interpreted cautiously and verified in studies involving larger cohorts of individuals with MS. Finally, measuring physical activity using a wrist-worn ActiGraph GT3X accelerometer may have limited the interpretation of the relationship between physical activity and locomotor functions, although this method reflected associations with the upper limb functions better, which is important in the context of the HGS assessment and the 9-HPT.

One of the chief strengths of this study was the use of validated tools for the assessment of functional fitness [22,70]. Walking functionality was assessed using the T25FW test, which is considered one of the most sensitive and recommended tools for the evaluation of mobility in persons with MS [71]. Furthermore, the procedure involved accelerometers for an objective measurement of physical activity, which are considered the most reliable method of assessing physical activity in this population [72]. An additional strength of the study was its comprehensiveness, as it included an assessment of each participant’s body composition, functional fitness, level of physical activity and diet. A multifactor regression analysis allowed us to investigate the relationship between the consumption frequency of protein-rich products, level of physical activity and low muscle mass (according to the criteria of sarcopenia). Each factor was presented as an independent variable associated with functional fitness, which allowed them to be compared with one another and to be interpreted in an easily comprehensible manner. 

## 5. Conclusions

The results of this study suggest that muscle mass is associated with physical fitness in persons with MS. A high level of physical activity may be associated with better maintenance of functional fitness, while more frequent consumption of protein-rich foods is associated with higher muscle mass necessary for effective locomotion. The findings should be interpreted with caution, as the cross-sectional and exploratory nature of the study allows for the identification of associations only and does not permit conclusions regarding causality. 

These findings provide a basis for future analyses aimed at verifying the direction and strength of the relationships between the studied variables using both cross-sectional and prospective approaches, with particular emphasis on the role of protein-rich foods in the diet of persons with MS at risk of sarcopenia.

Further prospective research is needed, including a comprehensive assessment of physical activity, diet, and muscle function, to better understand the relationships between muscle mass, dietary patterns, level of physical activity, and functional fitness in patients with MS.

## Figures and Tables

**Table 1:** Characteristics of participants in groups with different muscle masses.

	Normal ASMI (*N* = 81)		Low ASMI (*N* = 25)		
	*X* (*SD*)	*Me* (IQR)	*X* (*SD*)	*Me* (IQR)	
Sex					
Woman *N* (%)	73 (90.12)		10 (40.00)		0.001 ^A^
Men	8 (9.88)		15 (60.00)		
Type of SM					
Relapsing–remitting	71 (87.65)		22 (88.00)		0.978 ^A^
Primary progressive	4 (4.94)		1 (4.00)		
Secondary progressive	6 (7.41)		2 (8.00)		
Age (years)	43.51 (11.09)	44.0 (14.00)	39.72 (12.31)	41.00 (20.00)	0.192 ^B^
MS duration	10.38 (8.82)	8.0 (12.0)	9.85 (10.12)	8.0 (8.00)	0.459 ^B^
EDSS	2.66 (1.46)	2.50 (2.00)	2.32 (1.73)	2.00 (1.50)	0.093 ^B^
EDSS > 3.5 N (%)	71 (87.65)		22 (88.00)		0.963 ^A^
≤3.5	10 (12.35)		3 (12.00)		
Socio-economic situation					
Very good	21 (26.25)		5 (20.00)		0.505 ^A^
Good	7 (8.75)		3 (12.00)		
Satisfactory	5 (6.25)		0 (0.00)		
Poor	47 (58.75)		17 (68.00)		
Physical activity and sedentary behaviour					
% in SB	61.85 (6.96)	62.10 (8.88)	62.55 (6.28)	61.16 (4.59)	0.656 ^C^
% in light PA	22.78 (3.80)	22.34 (3.81)	22.03 (3.30)	22.59 (4.07)	0.589 ^B^
% in MVPA	15.31 (5.43)	14.49 (6.43)	15.42 (4.95)	15.38 (6.12)	0.853 ^B^
Fitness (or functional fitness)					
T25FW (s)	5.89 (3.15)	4.95 (1.76)	7.83 (8.37)	4.72 (1.48)	0.836 ^B^
9-HPT, dominant hand	21.42 (6.33)	19.95 (4.51)	21.50 (5.65)	20.94 (4.36)	0.836 ^B^
9-HPT, non-dominant hand	22.65 (7.19)	21.00 (4.44)	22.30 (4.61)	21.17 (6.28)	0.756 ^B^
HGS, dominant hand (kg)	30.06 (9.45)	28.80 (11.60)	23.40 (7.27)	23.75 (8.00)	0.001 ^B^
HGS, non-dominant hand (kg)	27.40 (9.70)	26.10 (13.30)	22.08 (6.08)	20.60 (6.80)	0.006 ^B^
Protein-rich products	3.12 (1.40)	2.95 (1.70)	2.90 (1.37)	3.08 (2.38)	0.820 ^B^
Body components					
BMI	25.55 (4.69)	24.80 (6.20)	22.10 (4.38)	22.20 (3.60)	0.001 ^B^
BMI < 25 kg/m^2^ N (%)	41 (50.62)		22 (88.00)		0.001 ^A^
≥25kg/m^2^ N (%)	40 (49.38)		3 (22.00)		
FatM	20.84 (10.10)	19.30 (10.70)	17.05 (8.00)	15.20 (6.10)	0.044 ^B^
FatP	27.20 (8.76)	28.30 (9.40)	28.11 (7.69)	26.90 (6.20)	0.642 ^C^
PMM	49.95 (8.76)	47.50 (12.60)	40.29 (5.20)	39.80 (6.20)	0.001 ^B^
Arms PMM	5.19 (1.47)	4.70 (2.10)	3.76 (0.60)	3.80 (0.80)	0.001 ^B^
Legs PMM	16.07 (3.33)	15.00 (4.90)	12.81 (1.49)	12.70 (2.10)	0.001 ^B^
Trunk PMM	28.70 (4.08)	27.80 (5.70)	23.72 (3.20)	23.50 (3.90)	0.001 ^C^
FFM	52.60 (9.19)	50.00 (13.30)	42.45 (5.47)	41.90 (6.50)	0.001 ^B^
Arms FFM	5.46 (1.57)	4.90 (2.30)	3.96 (0.60)	4.00 (0.80)	0.001 ^B^
Legs FFM	16.54 (3.41)	15.50 (4.90)	13.19 (1.53)	13.10 (2.20)	0.001 ^B^
Trunk FFM	30.13 (4.25)	29.30 (6.00)	24.92 (3.37)	24.60 (4.10)	0.001 ^C^

*N*—number of patients; ^A^—chi-square test; ^B^—Mann–Whitney *U* test; ^C^—Student’s *t*-test; FFM—fat-free mass; PMM—predictive muscle mass; BMI—body mass index; HGS—handgrip strength; T25FW—Timed 25-Foot Walk test; 9-HPT—9-Hole Peg test; MS—multiple sclerosis; EDSS—Expanded Disability Status Scale; SB—sedentary behaviour; PA—physical activity; MVPA—moderate-to-vigorous physical activity; FatM—fat mass in kg; FatP—fat percentage; *X* (*SD*)—mean (standard deviation); *Me* (IQR)—median (interquartile range).

**Table 2:** Multivariable regression analysis for T25FW, 9-HPT and HGS, where ASMI, MVPA and consumption frequency of protein-rich products were the predictors.

Predictors	Categories	T25FT			
		B	95% CI	SE	*p*
ASMI	-	0.14	−0.43; 0.02	0.14	0.299
% in MVPA *Ref. T1*	T2	−0.04	−0.43; 0.44	0.20	0.822
	T3	0.06	−0.45; 0.43	0.20	0.751
Protein consumption (frequency/day)	-	−0.22	−0.43; −0.02	0.10	**0.033**
		Dominant HGS			
ASMI	-	4.57	3.25; 5.90	0.68	**0.001**
% in MVPA *Ref. T1*	T2	−2.13	−4.12; −0.13	1.02	**0.037**
	T3	0.63	−1.39; 2.66	1.03	0.540
Protein consumption (frequency/day)		−0.06	−1.08; 0.96	0.52	0.909
		Non-dominant HGS			
ASMI	-	3.88	2.59; 5.16	0.66	**0.001**
% in MVPA *Ref. T1*	T2	−3.11	−5.08; −1.16	1.00	**0.015**
	T3	2.30	0.33; 4.26	1.00	**0.022**
Protein consumption (frequency/day)	-	−0.30	−1.31; 0.70	0.51	0.551
9-HPT non-dominant hand					
ASMI	-	0.17	−0.70; 1.05	0.45	0.696
% in MVPA *Ref. T1*	T2	−0.66	−1.96; 0.64	0.66	0.318
	T3	−1.73	−3.05; −0.40	0.67	**0.010**
Protein consumption (frequency/day)	-	0.19	−0.48; 0.85	0.34	0.577
9-HPT dominant hand					
ASMI	-	0.21	−0.57; 1.10	0.65	0.532
% in MVPA *Ref. T1*	T2	−0.66	−1.90; 0.59	0.63	0.301
	T3	−1.41	−2.68; −0.15	0.65	**0.029**
Protein consumption (frequency/day)	-	0.04	−0.60; 0.67	0.32	0.902

ASMI—Appendicular Skeletal Muscle Mass Index; MVPA—moderate-to-vigorous physical activity; 9-HPT—9-Hole Peg Test; T25FW—Timed 25-Foot Walk Test; HGS—handgrip strength; T1–T3—terciles.

## Data Availability

Data are available from the corresponding author upon reasonable request.

## Abbreviations

The following abbreviations are used in this manuscript:

MS	Multiple Sclerosis
ASMI	Appendicular Skeletal Muscle Index
BMI	Body Mass Index
EDSS	Expanded Disability Status Scale
FatM	Fat Mass
FFM	Fat-Free Mass
GS	Gait Speed
HGS	Handgrip Strength
IQR	Interquartile Range
MVPA	Moderate-to-Vigorous Physical Activity
Me	Median
N	Number of Patients
PA	Physical Activity
PMM	Predictive Muscle Mass
RRMS	Relapsing–Remitting Multiple Sclerosis
SB	Sedentary Behaviour
SD	Standard Deviation
T25FW	Timed 25-Foot Walking Test
T	Tertile
X	Mean
9-HPT	9-Hole Peg Test
